# Intergenerational Taekwondo Program: A Narrative Review and Practical Intervention Proposal

**DOI:** 10.3390/ijerph19095247

**Published:** 2022-04-26

**Authors:** Yongseop Kim, Junhyoung Kim, Jung-Min Lee, Dong-Chul Seo, Hyun Chul Jung

**Affiliations:** 1 Department of Health & Wellness Design, School of Public Health, Indiana University, 1025 E 7th Street #111, Bloomington, IN 47405, USA; yk67@iu.edu (Y.K.); kim9@iu.edu (J.K.); 2 Department of Physical Education, College of Physical Education, Kyung Hee University-Global Campus, 1732 Deokyoungdaero, Giheung-gu, Yongin-si 17014, Gyeonggi-do, Korea; jungminlee@khu.ac.kr; 3 Sports Science Research Center, Kyung Hee University-Global Campus, 1732 Deokyoungdaero, Giheung-gu, Yongin-si 17014, Gyeonggi-do, Korea; 4 Department of Applied Health Science, School of Public Health, Indiana University, 1025 E 7th Street #111, Bloomington, IN 47405, USA; seo@iu.edu; 5 Department of Coaching, College of Physical Education, Kyung Hee University-Global Campus, 1732 Deokyoungdaero, Giheung-gu, Yongin-si 17014, Gyeonggi-do, Korea

**Keywords:** intergenerational program, physical activity, taekwondo, social relation

## Abstract

Taekwondo is a modernized martial art that includes various combinations of hand and kicking techniques and core values of Taekwondo philosophy such as courtesy, mutual respect, and self-control. Physical inactivity is highly prevalent among older adults and is a major contributor to health-related problems. Intergenerational physical activity programs are used as an effective tool to make a positive connection between generations and provide additional health benefits for both generations. This review study aimed to examine the theories of intergenerational physical activity programs and propose the Intergenerational Taekwondo Program (ITP). Various theories such as the transtheoretical model, contact theory, social capital theory, situated learning theory, human development theory, personality theory, and whole-person wellness model have been adopted in intergenerational physical activity programs. Our review suggests that to develop the Intergenerational Taekwondo Program, instructors should (1) establishing common goals, (2) understand differences in physical and mental abilities, and (3) offer incentives to encourage participants in physical activity programs. The proposed ITP program has the potential to not just provide unique inherent values and improving physical functions, but also to form generational connections.

## 1. Introduction

Physical inactivity is a major contributor to health-related problems and affects both elderly and young adults in the United States (U.S.). Older adults who are over 65 years old show the highest prevalence of physical inactivity [1]. It has been suggested that physical inactivity is a contributory factor to physical limitations, psychological disorders (e.g., loneliness, depression, and anxiety), and cognitive impairments among older adults [1,2,3]. In particular, physical inactivity is one of the primary risk factors leading to falls as well as depressive symptoms [4,5]. There is strong support that physical activity is an effective intervention that can prevent falls and improve both mental health and quality of life in older adults [6,7].

Older adults are experiencing diminished engagement in social interaction and meaningful activities [8]. These common phenomena cause negative psychological symptoms that may influence dementia and lower quality of life for these populations. Moreover, the view of aging, which includes pessimistic viewpoints and bias towards older adults, is universal in our society [9]. Several studies indicated that society has more negative perspectives of older adults. Dedeli et al. [10] investigated the attitudes towards aging and perceptions of elder abuse in working and professional groups. This study showed older adults faced challenges in socializing with younger-generation community members because of their negative viewpoint and prejudices on the ability of older adults.

Intergenerational programs have been advocated as a means of promoting health and wellbeing among both the young and the old [11,12]. Given the significance of increasing physical activity, enhancing mental health, and reducing ageism, this study proposes to take a novel and innovative approach where both older adults and the young generation are provided an opportunity to engage in the mind–body exercise program together. Prior studies have shown that intergenerational programs help decrease the risk of social isolation and loneliness for older adults and provide a greater sense of meaning [11,12]. From younger participants’ perspectives, intergenerational programs allow adolescents and young adults to create meaningful relationships with older adults, develop interpersonal skills, and value their community engagement [13,14]. Minghetti et al. [15] reported that physical fitness and social support of both younger and older participants were improved following 25 weeks of an intergenerational dynamic balance exercise program. Another study also reported that the levels of depression and anxiety were decreased after engaging in 12 weeks of intergenerational interaction programs [16].

Taekwondo, a traditional Korean martial art and mind–body exercise, can be applied as a novel strategy of promoting physical, social, and psychological benefits for both older and young generations. Taekwondo is a martial art that emphasizes a unified relationship among body, mind, and spirit [17]. Taekwondo involves dynamics of physical movements such as various stances, punching, kicking, and self-defense techniques as fun components. It also emphasizes self-discipline, self-control, composure, stress management, and respect for opponents. Research has found that Taekwondo participants improve not only physical skills, such as strength, speed, flexibility, and balance, but also their self-regulation, self-discipline, and respect for others [18,19]. Moreover, multiple studies have shown that adolescents and young adults who participated in Taekwondo experienced increased enjoyment, youth development, positive self-image, and emotional stability. Thus, Taekwondo helps young adults to reduce perceived stress and improve psychological wellbeing [18,19]. This narrative review provides an overview of the intergenerational programs and summarizes the physical activity programs applied between younger and older generations. Upon the review of previous studies, we suggest that the utilization of Intergenerational Taekwondo Programs may have some capacity to increase the overall health in both young and older generations.

## 2. Intergenerational Theory and Exercise Programs

### 2.1. Intergenerational Program Theory

Several theories have been used for elaborating the importance of intergenerational activities, and highlighting focal points of their components, which explain older adults’ social, physical, psychological health throughout their lifespans. Seven related intergenerational theories were identified from studies that have been utilized for intergenerational activities such as arts, exercise, and recreational physical activity programs regarding improving health benefits for both generations

▪ *Transtheoretical Model (TTM)—*TTM refers to six stages of changes that individuals move through in terms of starting new behaviors or participating in activities. The six stages include precontemplation, contemplation, determination, action, relapse, and maintenance. Each stage has different spans depending on individuals’ cognitive, psychological levels [20].
▪ *Contact Theory—*According to contact theory, prejudice arises from generalizations and misrepresentations about a group of people based on inaccurate or insufficient information. The primary concept is that when one learns more about a group of individuals, prejudice may be lessened [21].
▪ *Social Capital Theory—*Social capital theory maintains that social relationships have the power to influence individuals’ resources, which enables the development and accumulation of human capital. Social capital theory examines how social relationships can positively impact on individual and organization levels beyond the original context of its making. The great value of social capital is in its potential to shift and facilitate other types of capital value for both individuals and organizations. It is only beneficial when social capital is mobilized, and as a form of system, it grows on itself.
▪ *Situated and Contextualized Learning Theory—*Situated and contextualized learning theory posits that individuals who acquire professional skills through learning within situational contexts will acquire membership in a community of practice. Situated learning provides opportunities to experience problem-solving and hands-on experience for practitioners. Lave and Wenger [21] argue that situated learning theory enables individuals to focus on the forms of social engagement instead of being concerned with what cognitive processes and conceptual structures are required.
▪ *Human Development Theory—*Erikson argued that personality development occurs in a predetermined order shown by eight stages of psychosocial development, from infancy to adulthood. Challenges individuals might experience during these stages may cause positive or negative outcomes for the development of personality. The psychosocial and educational benefits of connection between older and younger individuals are emphasized in the components of intergenerational activities.
▪ *Personality Theory—* Many scientists have stressed the significance of unconscious instinct in personality development and formation. Erik believed that the social element is critical for the development of personality, and it forms over the course of the entire lifespan. Some studies emphasized the importance of others in interpersonal relationships and in the construction of personality.
▪ *Whole Person Wellness Model (WPWM*)—WPWM refers to the combination of multiple aspects of health-related beliefs and meaningful activities for the individual. The WPWM consists of six dimensions, which include intellectual, social, emotional, vocational, spiritual, and physical. The objective of the WPWM is that individuals find balance among six elements of daily life activities. The main objective of the model is to seek an element of life activities that contributes to enhancing functioning and quality of life.

### 2.2. Intergenerational Exercise or Physical Activity Program

A total number of 13 studies that cover a range of applications were selected as intergenerational physical activity/exercise programs (Table 1). These programs were chosen based on the type and duration of interventions, as well as the engagement of both generations in physical activity at the same time. Selective intergenerational exercise and physical activity programs are described in Table 1. The age of older adult participants ranged from 50 to 96, the age of young generations ranged between 2 and 7, and young adults were 18 to 30 years old. Two studies used Mini-Mental State Examination (MMSE) [22,23] and Physical Activity Readiness Questionnaires (PARQ) [24,25] to determine sample eligibility. Ten out of thirteen studies included only older adults who could voluntarily participate in physical activity without physical and psychological limitations. Nine out of thirteen studies were conducted in community-based institutions such as community centers [23,26,27], universities [24,25,28], or leisure/recreational centers [29,30,31] while only two studies included older adults residing in facilities [14,31].

The 13 proposed intergenerational activity programs varied depending on the duration, types, outcomes, and characteristics of participation. Seven programs were short-term interventions and lasted between four weeks and 3 months, and long-term interventions lasted 5 years. Although it was challenging to compare varied physical activity in many aspects, it was possible to identify some program features that were applied to older adults. These were sorted into three different categories: physical function improvement, psychological health benefits, and social and emotional development. For example, Minghetti et al. [14] focused on physical functions such as balance, lower-limb strength, and cardiovascular health after receiving the dynamic balance exercise, while Choi and Sohng [22] compared loneliness and depression levels of both generations following the intergenerational exchange program.

A variety of self-reported and proxy measures were implicated across the studies. Eleven studies applied self-reported measures that detect perceptions of psychological wellbeing, self-efficacy, depression, and the level of physical activity. Three studies that measured physical functions such as gait speed, gross motor skills, and balance were conducted by health professionals or staff in institutions. Only one study presented contradictory results. Mosor et al. [31] reported no significant changes in older generations’ self-efficacy and activity engagement, while the younger generation showed higher engagement and longer duration of attention and listening skills following the intervention. Most interventions demonstrated positive outcomes of interventions among elderly groups.

There are several benefits to both physical and social health throughout participation in intergenerational programs. The first and most important is that intergenerational activities are beneficial for enhancing social health because they offer a sense of mutual links between two generations, as evidenced by precise theory and practice. Louise Douse et al. [20] found that older adults who engaged in the activity had increased social integration and positive emotions, and Minghetti et al. [15] and Choi and Sohng [22] reported that older adults had enhanced psychological health. Positive factors resulting from mutual participation are also essential for maintaining continual physical activity [23].

The second is that intergenerational activities that incorporated the learning of communication skills and decision-making processes are beneficial. Participants in the program, as Kim [23] mentioned, learn how to share their thoughts and feelings in a natural setting, which varies from conventional one-sided communication programs in that it gives a variety of opportunities for communication. In this regard, the intergenerational taekwondo program may be considered a good example because it allows partners to set goals and adapt and supplement each other’s posture.

The final is that, if the literature’s impression is correct, intergenerational activity programs can help improve cognitive capacities. We identified publications that support the idea of using activities to improve memory and attention span. Both generations demonstrated enhanced attention and listening, as well as emotional expression, according to Morsor et al. [31]. The results of the studies showed that continual intergenerational activities have a good impact on the participants’ cognitive characteristics.

## 3. Development of Intergenerational Taekwondo Program

### 3.1. Taekwondo Culture

Taekwondo, a traditional form of Korean martial arts, literally translates to “the way of feet and hands,” and its origins began in the Korean peninsula about two thousand years ago. As with all other disciplines, it is essential for the Taekwondo trainers to be familiar with its five tenets. Although many scholars argue that Taekwondo tenets and principles should be reestablished as they solely focus on spiritual aspects, [32] and there are different principles based on types of training [33,34], nevertheless, General Choi Hong-hee’s five tenets of Taekwondo are still maintained as the principles that encompass the spirit, culture, and concept of Taekwondo, and they are still being followed by many trainees around the world. The five tenets of Taekwondo are “Courtesy”, “Integrity”, “Perseverance”, “Self-Control”, and “Indomitable spirit”.

Some scholars assert that the national representative martial arts have great value in cultivating core values that are required of citizens [34,35,36]. Ryu [36] mentioned that in Japan, the noble spirit was sublimed into the universal spirit of Japan in terms of the “honor and character-building” of Samurai, and its concepts include righteousness, courtesy, loyalty, and self-control. Moreover, it is mandatory to learn these tenets through participating in martial arts with regular school curriculums. Moreover, Lee et al. [34] argued that practitioners learn different aspects of values according to the belt system in Taekwondo, which include “fairness”, “perseverance”, “harmony”, and “satisfaction”. In this respect, Taekwondo can play a significant role in cultivating significant values throughout Taekwondo training.

Among these principles, some scholars captured the significant culture of mutual respect in Taekwondo. According to Lim [35], children (mean age 12) who participated in Taekwondo for 6 months increased greeting, language, and public etiquette. In addition, the study showed that participants improved more on etiquette as they learned for longer periods. In addition, Kim and Yeo [37] argued that training Taekwondo is a process of learning normative attitude and morality, which are important for the role of individuals as members of society. This highlights the “courtesy” among the five tenets of Taekwondo.

For older adults, however, Taekwondo’s intrinsic value leads to continued engagement, resulting in values such as mutual respect, improved health perception, and improved quality of life. For example, Yang [38] demonstrated significant associations between Taekwondo participation and quality of life and perceived health among older adults. In this regard, according to Stebbins [39], “unique ethos” refers to the existence of differentiating ideas, values, feelings, or guiding beliefs that contribute to the creation of a distinct social environment and the establishment of a social network. Taekwondo’s unique culture encourages both older and younger generations to engage in the activity in a continuous and beneficial manner in this respect.

### 3.2. Intergenerational Taekwondo Program

We applied two theories that were used in previous intergenerational studies [22,24,25]. Choi and Sohng [22] employed the Contact Theory to reduce stereotypes of aging and develop health by engaging older adults and younger generations in intergenerational physical activity programs. A previous study reported that interventions using five components in Contact Theory (support from authority, common goal, opportunity for friendship, cooperation, and equal group status) were more useful compared to non-theory-driven interventions [40]. Choi and Sohng [22] used a community-based intergenerational exchange program on older adults to improve older adults’ health-related quality of life, loneliness, depression, and walking speed. They utilized five strategies in the intervention, where a single component counts for each stage. For example, “common goal” refers to establishing a detailed goal of making traditional artwork planned for each session, and “equal group status” indicates engaging in a part of a traditional play that does not have a distinctive role in the activities. In our proposed Intergenerational TKD Program, both generations share goals for each week’s activity, such as learning high blocking, basic stances, which can be a part of common goals in this theory. In addition, for the part of self-defense, both generations teach each other and conduct what they have learned from the class, which is the “equal group status” component of the theory.

In addition, Strand et al. [24] and Sowle [25] focused on five components of the Whole Person Wellness Model in intergenerational activity. The WPWM is a comprehensive approach to successful aging that is being applied in many different senior living community-wellness programs [26]. This concept incorporates personal wellness components including (1) social, (2) spiritual, (3) physical, (4) emotional, (5) intellectual, and (6) occupational/vocational, which were used in the Living Intergenerational Fitness and Exercise (LIFE) Program [24,25]. In the program, WPWM was applied to support participants’ wellness concepts including self-efficacy, self-responsibility, and multiple dimensions of wellness. In the study done by Sowle [25], exergaming physical activity matched the composition of physical, social, and emotional components of the WPWM. In the proposed program, greetings and socializing in the introductory part cover the “social” component of the model, and partner self-defense practice comprises the physical, emotional, intellectual, and vocational components of the model.

In particular, we highlight activities made by a mutual contact and that apply the elements contained in WPWM. Table 2 shows how these two theories were applied to the development of the intergenerational Taekwondo program.

### 3.3. Lessons Learned: Recommendations for Instructors and Health Care Services

As much of the above empirical evidence demonstrated, there are significant health benefits from participating in intergenerational activities for both younger generations and older adults. There are some recommendations to encourage participation and improve the retention rate of continuous participation. Kim [23] mentioned that intergenerational activities provide mutual responsibilities that largely impact older adults’ engagement and participation. However, there are no ample studies that suggest the key elements for sustainable performance in intergenerational physical activities. Thus, the current study proposes a few strategies for instructors and health care services that could help both generations engage in the intergeneration Taekwondo programs more effectively and successfully.

First, establishing common goals. As Choi and Sohng [22] noted, establishing common goals based on their needs and capability is a key component of making strong bonds between partners. Taekwondo has learning goals corresponding to the color of the trainee’s belt, such as low block and front kick. These characteristics may serve as effective motivators for trainees to participate in the activity. In order to set up goals for both generations, instructors facilitate a small session focused on socializing and creating rapport before starting the program. It is important to emphasize training-related concepts that both generations can apply within the program and achieve together. For example, this includes the two agreeing on the importance of continuous physical activity and expressing their willingness to participate in the program.

Secondly, accommodating differences in ability. Some studies asserted that the lack of interest in participation in programs due to different physical functions between generations was one of the important factors affecting continuing participation [16]. When working with both generations, instructors may need to accommodate differences in abilities such as strength, flexibilities, or body coordination. While some Taekwondo movements help to improve overall health and change the level of physical function over the life course, students may be more interested in improving techniques and fun aspects. Instructors may need to consider accommodating differences in abilities for both generations.

Thirdly, offering incentives is an effective means to encourage participants to engage in physical activity programs. Many studies found that offering incentives increased participants’ physical activity at multiple levels [41,42]. According to Capps and Harkey [43], approximately 70% of people were more engaged in physical activity when financial incentives were provided by team-based participation rates. A wide variety of incentives helps participants to maintain participation rates over a long period. The Taekwondo belt system may be used as an effective means of motivating participants when they engage in activity with their partner.

Lastly, encouraging both generations to help one another as often as possible. Kim [23] mentioned that in the intergenerational exercise program, participants were more engaged in physical activities and utilized fine and gross motor skills while they helped to reach goals together. Taekwondo movement requires a sense of balance and intensive physical coordination. Physical function could be improved while guiding participants to cooperate to perform standing or blocking movements during the training.

## 4. Conclusions

Many practitioners and researchers have highlighted the importance of intergenerational activity programs, as they appear to have positive health benefits for both generations. This conceptual approach for the development of an Intergenerational Taekwondo Program demonstrates an effective means of improving health for both generations. The suggested program involves a combination of unique values of Taekwondo such as courtesy, mutual respect, and self-control, and a variety of movements that enhance participants’ physical functions. This form of social-intervention physical activity program allows participants to transfer and exchange their knowledge, skills, and abilities. Moreover, mutual bonds between participants could be the main factor in maintaining physical activity participation in the Intergenerational Taekwondo Program. Future research is needed to shed light on the implication of the Intergenerational Taekwondo Program for older adults living in different settings (including assisted-living community, independent living, or memory care unit). Furthermore, in a society where the number of older adults is rapidly increasing, long-term policies for collaboration with local communities that could extend physical activities between generations appear to be necessary.

## Figures and Tables

**Table 1:** Summary of intergenerational physical activity intervention studies.

Reference	Study Population	Setting	Intervention Content		Outcome Measurement		Effectiveness		Intergenerational PA Components
			Length	Key Activities	Instrument	Content	YG	EG	
Louise Douse et al. (2020)	25 students (age 14 y) 11 older adults (age average 82 y)	University	11 weeks	Generations Dancing	Basic Psychological Needs Satisfaction Scale Positive and Negative Affect Scale (PANAS) Social Wellbeing Scale Basic Psychological Needs Satisfaction Scale	Basic Needs Affect Social Wellbeing	N/A	Social Integration ↑ Social Acceptance ↑ Positive Emotions (enjoyment, excitement, confidence, pride) ↑	N/A
Eun Hae Kim (2021)	17 younger adults (age 18–25) 34 older adults (age 65+)	Community center	8 weeks	Tai Chi Chair Yoga	Berlin Social Support Scale Intention and retention survey questions (scale 1–7)	A sense of social support Intention to continue Retention Sociodemographic information	Sense of social support -	Sense of social support—Intention to continue ↑ Retention ↓	N/A
Minghetti et al. (2021)	72 children (age 4–6) 62 older adults (age 65+)	Nursing home	25 weeks	Dynamic Balance exercise	YG: Test of Gross Motor Development 2 (TGMD-2) KOMPIK questionnaire EG: Short Physical Performance Battery (SPPB) Oscillometric Mobil-O-Graph Assessment of Quality of Life 8 Dimensions (AQol-8D)	YG: Gross motor skills Social-emotional skills EG: Balance, gait speed, lower-limb strength Cardiovascular health Psychosocial wellbeing and quality of life	Physical performance ↑ (jump power, hand grip strength) Social-emotional skills ↑ (small to moderate)	Physical performance ↑ Cardiovascular health ↑ Psychological wellbeing and quality of life ↑	N/A
Mosor et al. (2019)	78 children (age 2–7) 93 older adults (age 54–96)	Geriatric institution	20 weeks	Intergenerational contact activity	Facial expression Engagement/behavior Intergenerational interaction Observed self-efficacy	Active engagement Self-efficacy	Active engagement (Facial expression: Happy/smiling) ↑ Paying attention/Listening ↑ Initiating intergenerational interaction ↑ Self-efficacy—no changes	No significant changes	N/A
Ebrahimi et al. (2019)	60 youth (mean age 22) 175 older adults (age 60+)	Community service center	12 weeks	Intergenerational interaction program Yoga Control group	General Health Questionnaire (GHQ-28)	Depression Somatic symptoms Social Function symptoms Anxiety and depression	N/A	Somatic symptoms ↓ Social Function symptoms ↓ Anxiety and sleep disorder ↓ General health ↑	Interaction includes any younger person’s active involvement with an elderly person and fulfilling the wishes of the elderly individually for 45 min
Sowle (2015)	Younger adults (age 14–28) 265 older adults (age 60+)	University	24 weeks	Exergame Interactive games	Cancer Prevention Research Center Exercise The Self-Efficacy for Exercise Scale	Self-reported activity level Physical activity self-efficacy	N/A	Physical activity level ↑ Overall physical activity self-efficacy- Self-efficacy for overcoming physical activity barriers ↑	Using Microsoft Kinetics (detect the participants’ motion through a sensor). Participating in Wii soccer, volleyball, track and field, bowling, boxing, and table tennis. Two participants (or younger adult leaders) lead the program and the others follow the motions.
Choi and Sohng (2018)	70 Children (age 4–5) 88 older adults (65+)	Senior Center	8 Weeks	Intergenerational Exchange Program	Health-related quality of Life (HRQoL) UCLA Loneliness Scale Geriatric Depression Scale Short Form (GDSSF) 4-min walking test Learning-related social skills (McCelland and Morrison)	Health-related quality of life Loneliness Depression Walking speed Social skills	Learning-related social skills ↑	Quality of life ↑ Loneliness ↓ Depression ↓ Gait speed-	Sharing ideas about an appropriate frequency of programs, places, and things should be considered within the program. Utilizing Contact theories 5 strategies Setting up common goals between older adults and children At the end of the program, “Personal Friendship Chance” where older adults hug children with pinky finger.
Buonsenso et al. (2021)	140 Older adults (age 50–85)	Gymnastic group, Leisure Center	N/A	Intergenerational physical activity	Global physical activity questionnaire (GPAQ) Physical Activity Enjoyment Scale (PACES-Q) Physical Activity Enjoyment Scale for Intergenerational Physical Activity (PACES-INT)	Physical activity enjoyment Intergenerational Physical activity enjoyment	N/A	Intergenerational Physical activity enjoyment ↑	N/A
Friedman and Godfrey. (2008)	30 older adults 50 Children (age 3–5)	Jewish Community Elderly Center	5 years (20–30 min)	The intergenerational Exercise (Warm-up breathing, Warm-up and cool down stretching, balance, aerobic, free-weight)	N/A	N/A	- Sense of competition ↑ - Listening skills and attention span ↑ - Stereotype attitude change ↑	- Memory ↑ - Sense of competition ↑ - Anxiety and Depression↓ - Self-validation, Self-esteem ↑ - Lifelong exercise ↑ - Kinesthetic awareness ↑	- Children and adults arrange intermingled circle (fun and serious) - Adult-level PA - Older adults help students to realize to concentrate on exercise - Leader, older adults praise, encourage students - Breathe deeply together (finding a common rhythm) - EG and YG make shapes, patterns and provide balance test (strengthening exercise) ** - EG and YG hold each-others hand and raise lower legs (Balance) - Marching together (Aerobic exercise)
Rhodes et al. (2010)	107 families	Local Recreation Center	4 weeks	Intergenerational Family Physical Activity	Godin Leisure Time Exercise Questionnaire (GLTEQ) Theory of Planned Behavior Ajen (2002)	Leisure Time Physical Activity Intention Perceived Behavioral Control		-Family physical activity ↑	N/A
Perry and Weatherby (2011)	7 youth (8–14) 7 older adults (60–85)	Community Gym	8 weeks (60 min)	Intergenerational Tai Chi	Seven-Day Physical Activity Recall (7DPAR) The Physical Activity Enjoyment Scale (PACES)	Physical activity Enjoyment	Physical activity ↑ Enjoyment ↑	Physical activity ↑ Enjoyment ↑	- Skill building and provide informational messages, positive reinforcement (Confidence) - Paired YG and EG interactive exercise pose
Strand et al. (2014)	46 older adults (60–80)	University	25 weeks	The Living well through Intergenerational Fitness and Exercise (LIFE): Exergaming Wii Active	Stages of Change for Physical Activity Questionnaire.	Physical activity Perceived Physical Wellness	N/A	Satisfaction ↑ Physical activity ↑ (Among physically inactive rural older adults)	- 30 min of interactive games (e.g., icebreakers, storytelling activities, strategy and mind games, weekly to promote camaraderie, problem solving, and communication skills)
McConnell and Naylor (2016)	22 elementary (Grade 4/5) 9 older adults (>55)	Community center	12 weeks	Intergenerational Physical Activity Leadership (IPAL): Walking marathon Playground games Chair aerobic	Systems for Observing Physical and Leisure Activity in Youth (SOPLAY) Children’s perception of aging and elderly scale Intergenerational Observation Scale (IOS) Environmental responsibility and leadership scale	Physical activity and environmental context Intergenerational attitudes Intergenerational interaction Knowledge and confidence			- Older adults remind rules, encouraging students - Older adults taught how to do Chair aerobic exercise - Older adults help to facilitate physical activity

Note. YG: young generation; OG: older generation (check the abbreviation).

**Table 2:** Development of Intergenerational Taekwondo Program.

Stages	Activity	Duration (min.)	Program Contents	Photos	Contact Theory	Whole Person Wellness Model (WPWM)
Introduction	Socializing	5	Older adults and young adults having a conversation.	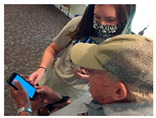	Support from authority Common goal Opportunity for friendship	Social
	Meditation	2–3				Spiritual
	Greetings	1	Standing: make a fist and keep back straight and look in front of the sight.	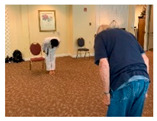		Social
Main Activity	Warm up	5	Walking around the room with line	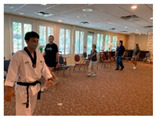		Physical
	Warm up Stretching	10	Upper-arm stretching	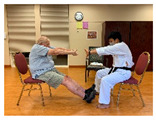	Equal group status Cooperation Support from authority Opportunity for friendship	Physical Emotional Intellectual Vocational
			Shoulder stretching	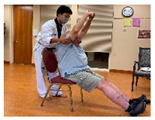		
			Neck stretching	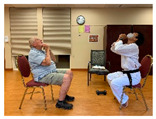		
			Side abdominal stretching	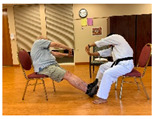		
			Single-leg stretching	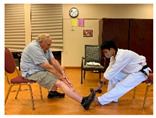		
			Isometric lower-body stretching	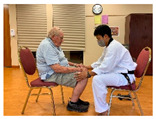		
	Taekwondo Practice (Individual)	10	Blocking (low, middle, high)	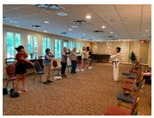	Support from authority	Physical
			Punching			
			Stances			
			Kicking (front and roundhouse)			
	Taekwondo Practice (Intergenerational)	15	Punching	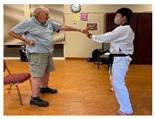	Equal group status Cooperation Support from authority Opportunity for friendship	Physical Emotional Intellectual Vocational
			Self-defense (low block)	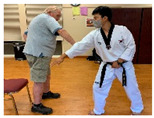		
			Self-defense (middle block)	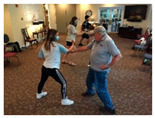		
Ending Greeting	Cool down Stretching	5	Whole-body stretching	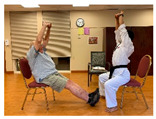		Physical
	Sharing feelings	5	Talk about your experience and feelings on Taekwondo activity		Opportunity for friendship Equal group status	Social Emotional
	Meditation	3				Spiritual

## Data Availability

Not applicable.
